# Different Changes of Risks for Stroke and Myocardial Infarction in Patients With Type 2 Diabetes in Hungary Between the Two Periods of 2001–2004 and 2010–2013

**DOI:** 10.3389/fendo.2019.00170

**Published:** 2019-03-21

**Authors:** Zoltan Kiss, György Rokszin, Zsolt Abonyi-Tóth, György Jermendy, Péter Kempler, István Wittmann

**Affiliations:** ^1^Second Department of Medicine and Nephrological Center, Faculty of Medicine, University of Pécs, Pécs, Hungary; ^2^RxTarget Ltd., Szolnok, Hungary; ^3^Department of Biomathematics and Informatics, University of Veterinary Medicine, Budapest, Hungary; ^4^Medical Department, Bajcsy-Zsilinszky Hospital, Budapest, Hungary; ^5^First Department of Medicine, Faculty of Medicine, Semmelweis University, Budapest, Hungary

**Keywords:** cardiovascular diseases, myocardial infarction, stroke, all-cause mortality, type 2 diabetes mellitus

## Abstract

**Objective:** In recent decades several studies reported significant changes in the mortality and morbidity of patients with type 2 diabetes. In this Hungarian nationwide study, we assessed the changes of mortality and cardiovascular risks comparing a group of patient diagnosed during the two periods of 2001–2004 and 2010–2013.

**Research Design and Methods:** We identified patients with type 2 diabetes recorded in the database of the Hungarian National Health Insurance Fund aiming to assess changes of risks for all-cause mortality, myocardial infarction, and stroke during the follow-up periods of 48 months.

**Results:** We included 274,109 patients with newly diagnosed type 2 diabetes between 2001 and 2004, while only 152,678 in the 2010–2013 period. The risk of all-cause mortality at the beginning of the follow-up was not different comparing 2010–2013 to 2001–2004 (HR 1.03), and a mild but significant decrease could be detected (HR 0.87) after 48 months. A lower risk for myocardial infarction was documented in the second month after the diagnosis of diabetes (HR 0.67), which remained constant during the study period and almost the same after 48 months (HR 0.73). On the contrary, our study verified a slightly increased risk for stroke close to the diagnosis [HR 1.06 (1.00–1.13)] and a decreased one [HR 0.72 (0.69–0.79)] at the end of the study.

**Conclusions:** An increased risk for stroke followed by a sharp decrease, and an unchanged risk for myocardial infarction in the Hungarian population with type 2 diabetes possibly resulted in a mild decrease of all-cause mortality between 2001 and 2013.

## Introduction

In recent years several reports have been published demonstrating significant changes in the risks of diabetes mortality and morbidity (1–4).

Between 1996 and 2009 in Canada and in the UK, the diabetes mortality rate ratio decreased from 1.90 to 1.51, as well as from 2.14 to 1.65, respectively (2). Between 2004 and 2012 in Israel the annual mortality rates among patients with diabetes decreased from 13.8/1000 to 10.7/1000 (*p* = 0.0002) (3). Based on the Tancredi report, a time interaction exists in Sweden as well, namely, an adjusted hazard ratio (HR) for death from all-cause among patients with diabetes was significantly lower in patients diagnosed in 2005 or later than before (HR 1.13 vs. 1.17 *p* = 0.004). Similar results were obtained for cardiovascular mortality (HR 1.11 vs. 1.19 *p* < 0.001) (4).

Norhammar assessed the risk of cardiovascular morbidity in the same Swedish population in a later period, between 2006 and 2013, showing a change in risk ratio for myocardial infarction (1.74–1.68 *p* = 0.037) and stroke (1.54–1.45 *p* = 0.003) in the type 2 diabetes group as compared to the non-diabetic Swedish population (5).

In another real-world-evidence-based study evaluating all-cause mortality and cardiovascular morbidity (myocardial infarction, stroke) risk of the U. S. population between 1990 and 2010, similar improvement in all cardiovascular parameters were confirmed. The largest decrease was detected in acute myocardial infarction [−67.8%; 95% confidence interval (CI), (−76.2 to −59.3)] and significant changes were also seen in the rate of stroke and amputations (−52.7 and −51.4%, respectively) (6).

We decided to examine the first 4 years of the last two decades to compare changes of morbidity and mortality risks in Hungarian patients with diabetes during the periods of 2001–2004 and 2010–2013.

## Methods and Study Design

This study was approved of by the Regional Research Ethics Committee of the Medical Center, University of Pécs, Hungary (study license number: 6962/2017.) and received no commercial sponsorship. The study protocol was also reviewed and confirmed by the National Health Insurance Fund (NHIF, ID: S04/161/2016).

**Figure 1:**
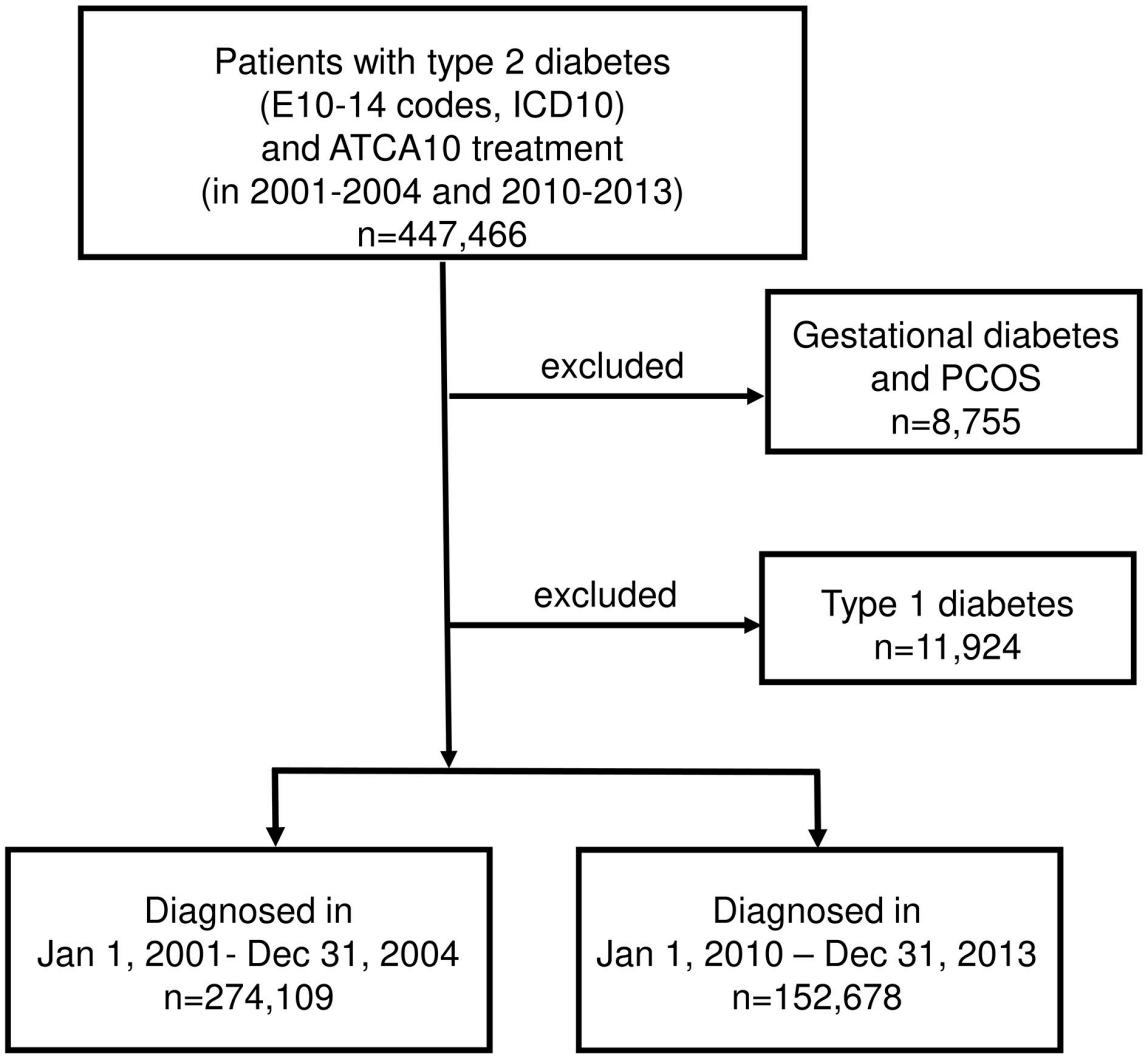
Enrollment and exclusions.

Data of type 2 diabetic patients undergoing antidiabetic therapy between January 1, 2001 and December 31, 2004 (*n* = 274,109) or between January 1, 2010 and December 31, 2013 (*n* = 152,678) were extracted from the database of the National Health Insurance Fund in the form of anonymized, aggregated patient data, and were considered as type 2 diabetes if receiving antidiabetic treatment (ATC A10), however not matching previously detailed and published criteria of type 1 diabetes (7) or not having an ICD code for PCOS and gestational diabetes (Figure 1).

The onset of diabetes was defined with the reference to the first occurrences of diabetes according to the International Classification of Diseases (ICD) code in the database, or at the start of the first antidiabetic treatment.

The data source included information on mortality from any causes, because this database does not differentiate between the causes of death, and provides data for cardiovascular complications of diabetes like incidence of myocardial infarction and stroke. Codes taken from the ICD (9 and 10th Revisions) were used supporting diagnoses of acute myocardial infarction, as well as stroke from Jan 1, 2000 onward.

All-cause mortality and cardiovascular morbidity (myocardial infarction: ICD-10 I21-24 in in-patient records and ischemic and hemorrhagic stroke: ICD-10 I61-63, G4630, G4640, G4580, G4590 in in-patient records) were assessed for 48 months in both groups, following the 2nd month after the onset of diabetes of both periods (Jan 1, 2001 and Jan 1, 2010). At least 1 year of screening period was conducted for all patients before the onset of diabetes, aiming to identify prior myocardial infarction or stroke event.

## Statistical Analysis

The Cox regression model was used to determine the hazard ratio of death, MI and stroke in patients diagnosed during 2001–2004 and 2010–2013. The model was adjusted for baseline differences of gender and age group (0–18, 19–30, 31–40, 41–50, 51–60, 61–70, 71 years). As the proportional hazard assumption was not satisfied in the model, we were using a time-dependent covariate: a linear function of time from diabetes onset for group 2010–2013 was included in the model. In this case, the Cox model estimates the hazard ratio as a continuous function of time.

The main advantage of this statistical approach is that we are able to estimate such differences in risk at any point of patient-life from diagnosis, comparing the periods of 2010–2013 to 2001–2004. Therefore, we are able to evaluate the risk around diagnosis-time, as well. Additionally, if we compare the same time points of the later phase of complications of diabetes, we are able to estimate the impact of diabetes care during a whole decade.

Although the MI time dependency was not significant, we integrated it into the model to show the mild observed changes.

Data were available for our analysis following October 31, 2014 as a follow-up period. We followed the patients from diagnosis to the event or death or till the end of a 48 months long follow-up period.

The risk during first month was much higher, probably because diabetes was diagnosed just at the occurrence of the event. To eliminate the possible bias, we started the follow-up after the first month following the diagnosis.

The analysis was performed with the use of R Software, version 3.4.2 (2017-09-28)—R Core Team (2017). R Foundation for Statistical Computing, Vienna, Austria.

## Results

Patient characteristics are shown in Table 1. The patients of the two time-periods differed in all parameters except for the prevalence of prior myocardial infarction.

**Table 1:** Characteristics of patients, mean ± SD.

	**Type 2 diabetes 2001–2004**	**Type 2 diabetes 2010–2013**	***p*-value**
Population (*n*)	274.109	152.678	*p* < 0.0001
Age (years)	59.33 (±12.43)	59.43 (±2.92)	*p* = 0.0088
Female - no. (%)	145.715 (53.2)	79.238 (51.9)	*p* < 0.0001
Age (years)	60.74 (±12.61)	60.89 (±13.70)	*p* = 0.0127
Male - no. (%)	128.394 (46.8)	73.440 (48.1)	*p* < 0.0001
Age (years)	57.72 (±12.01)	57.9 (±11.82)	*p* = 0.0094
Prior Myocardial Infarction (%)	1.427 (0.5)	814 (0.5)	*p* = 0.9608
Prior Stroke (%)	2.989 (1.1)	2.599 (1.7)	*p* < 0.0001

**Figure 2:**
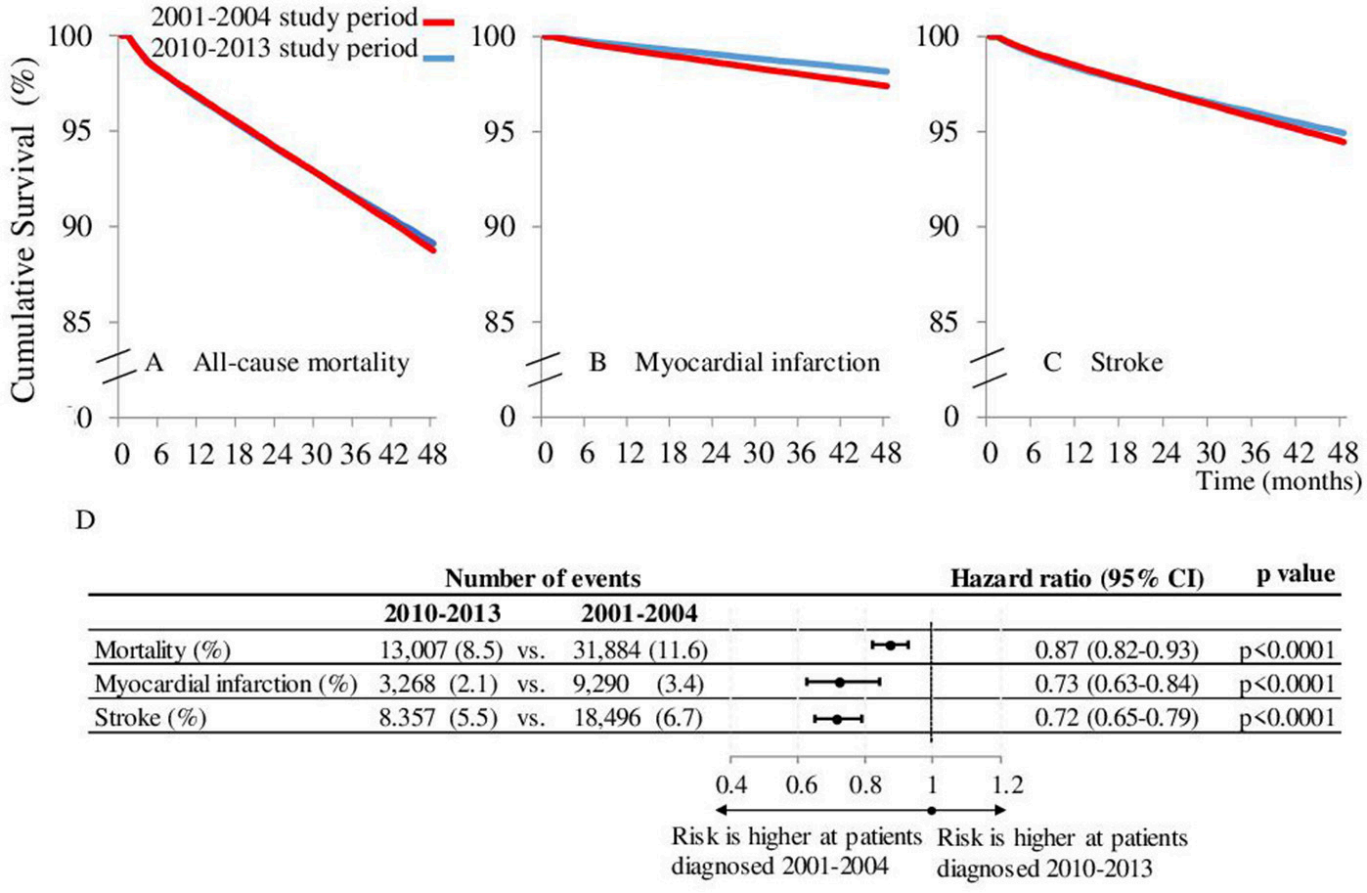
Kaplan-Meier mortality. **(A)** myocardial infarction **(B)**, and stroke **(C)** free survival curve comparing patients diagnosed between 2010-2013 to those diagnosed between 2001-2004 during 48 months survival period. **(D)** Represents the hazard ratios of the events after 48 months of the diabetes onset.

We recorded 31,884 cases of death out of 274,109 (11.63%) in the type 2 diabetes group between 2001 and 2004, whereas between 2010 and 2013, 13,007 out of 152,678 patients (8.52%) were identified by the end of the 48 months study period (Figure 2A).

Using a Cox regression analysis, comparing the periods of 2001–2004 and 2010–2013, the risk of all-cause mortality was not different in the second month after the diabetes onset [HR 1.03 (0.99–1.07); *p* = 0.2083], however after 48 months of follow-up the difference was significant [HR 0.87 (0.82–0.93); *p* < 0.0001; Figure 2].

**Figure 3:**
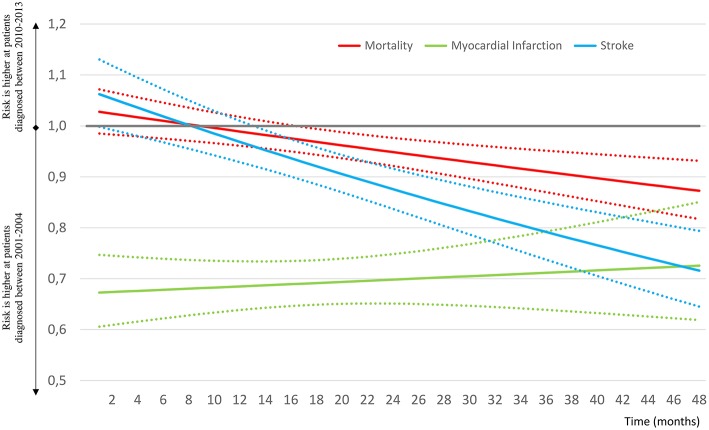
Changes of hazard ratios of all-cause mortality, myocardial infarction and stroke by month from diagnosis, comparing type 2 diabetes patients diagnosed during 2010–2013 vs. 2001–2004. The continuous lines represent the mean of HR, the dotted lines the 95% CI values.

Altogether, 9,220 and 3,268 cases of myocardial infarction and 18,496 and 8,357 stroke events were recorded during the 2001–2004 and 2010–2013 study periods (Figures 2B–D). We detected a lower risk for myocardial infarction in the second month [HR 0.67(0.61–0.74); *p* < 0.0001]. It was almost the same after 48 months [HR 0.73 (0.63–0.84) *p* < 0.001]. In the case of stroke the risk was higher in the 2010–2013 period in the second month (HR 1.06 (1.00–1.12); *p* = 0.0354) and appeared to be lower after 48 months (HR 0.72 (0.65–0.79); *p* < 0.0001). Hazard ratios at the start and at the end of study may not exactly reflect the nature of changes. Therefore, the data for HR-s for the whole follow-up period are presented as continuous variables, as well (Figure 3). Indeed, no breaks were found in the curves, rather a continuous decrease in respect of mortality and stroke HR-s, whereas we found an unchanged curve for myocardial infarction. The decrease of HR of mortality started at 1.03 (0.99–1.07) in the second month after the diabetes onset and crossed the fast dropping line of stroke at about the 8th month. The HR of stroke in the second month was 1.06 (1.00–1.13) and due to the high risk reduction of stroke its relative risk got close to that of myocardial infarction at the end of the follow-up period. The curve of myocardial infarction was unchanged during the study period [HR at the second month 0.67 (0.61–0.75) and at 48 months 0.73 (0.62–0.85)]. The absolute hazard ratio reduction per 48 months of study period was 0.16 concerning mortality and 0.35 for stroke, whereas the absolute hazard ratio's increase was 0.05 for the myocardial infarction, which was not significant (*p* = 0.6418).

## Conclusions

The aim of our study was to assess mortality and cardiovascular risks in a Hungarian population of type 2 diabetic patients, comparing two time periods of 2001–2004 and 2010–2014.

The two main findings of our study can be summarized as follows:

- At the start of the follow-up, in 2010, the risk for all-cause-mortality was the same as in 2001. During the follow-up, there was a slow decrease of the risk for all-cause-mortality in the period of 2010–2014 compared to that of 2001–2004.
- Surprisingly, 10 years after the start of the follow-up period of 2001–2004 an increased risk for stroke could be identified in 2010. During the follow-up, a fast decrease of stroke risk could be documented. As expected, the risk for myocardial infarction was lower in 2010 than in 2001. The slope of the risk-curve of myocardial infarction was unchanged during the study period.

Due to our study design, there are no comparable data in previous papers about all-cause mortality, myocardial infarction, and stroke. According to our method, not the whole available (2001–2013) dataset had to be used but just a part of it (from 2001 to 2004 and from 2010 to 2013) as shown in Figure 1. However, this might be considered as a limitation of our study, as it may not be known what happened to the parameters studied in the period between 2004 and 2010.

Indeed, our data reveal that the relative risk of stroke increased (6.2%) between 2004 and 2010 in the Hungarian population with type 2 diabetes, which resulted in a significantly higher risk at the diabetes onset in 2010 compared to 2001. The explanation of this increase remains unknown, however, this surprising evidence needs further assessment, even in the non-diabetic Hungarian population.

Similarly to other papers (4–6, 8), in our study we were able to detect a decreased risk for myocardial infarction at the time of the diagnosis of type 2 diabetes between 2001 and 2010 (risk reduction was 32.7%, Figure 2D), which did not change significantly during the 48 month follow-up period.

Recently published comprehensive analysis of population-based studies from several countries including the UK, USA, Canada, and South Korea, reported a significant risk reduction of stroke and myocardial infarction in the last two decades in parallel with a relevant decrease of diabetes mortality. Harding explains these declines in the rates of diabetes complications with multifactorial causes, improving relevant risk factors of the population and development of prevention strategies, CVD treatment procedures and treatment options (9).

The increased risk of stroke and the decreased risk of myocardial infarction might result in the unchanged risk of all-cause mortality at the diagnosis of diabetes, comparing the years 2001 and 2010. The significant drop of risk of stroke and the continuously low risk of myocardial infarction during the follow-up might be responsible for the mild amelioration of all-cause mortality risk at the 48th month.

Differences of the populations investigated (2001–2004 *n* = 274,109 vs. 2010–2013 *n* = 152,678) resulted from the decreasing trend of the incidence of diabetes in Hungary (10). The figures of newly diagnosed patients were 75,750, 65,600, 64,800, 65,100 during 2001–2004 and 39,700, 33,500, 29,900, 33,700 in the 2010–2013 period.

Using a screening period for stroke and myocardial infarction from January 1, 2000 may lead to a certain level of bias, as patients diagnosed later with T2DM have a longer period for prior event screening. Nevertheless, prior events do not constitute a part of any statistical analyses for the 48 months long follow-up period.

In conclusion, as a novel observation, our study has provided evidence that the risk for stroke in the Hungarian population with type 2 diabetes increased between 2004 and 2010, whereas the risk for myocardial infarction remained unchanged during the same period. Possibly as a results of these changes, a mild amelioration of the risk of all-cause mortality has been documented.

## Author Contributions

ZK participated in data collection. GR participated in data collection, prepared figures, and tables. ZA-T prepared figures and tables, performed the statistical analysis. GJ and PK wrote the manuscript and participated in the interpretation of data. IW designed the study, wrote the manuscript, and lead the author teamwork.

### Conflict of Interest Statement

GR and ZA-T are employed by RxTarget Ltd. The funder played no role in the study design, the collection, analysis or interpretation of data, the writing of this paper or the decision to submit it for publication. The remaining authors declare that the research was conducted in the absence of any commercial or financial relationships that could be construed as a potential conflict of interest.
